# Comprehensive Analysis of Wild Rice Mitochondrial Genomes Reveals Structural Variation, Repeat Dynamics, and the Evolution of *orf182*

**DOI:** 10.3390/plants15071111

**Published:** 2026-04-03

**Authors:** Weixiong Long, Jie Wang, Lihua Luo, Lujian Zhou, Wei Chen, Laiyang Luo, Weibiao Xu, Yonghui Li, Longan Yan, Yaohui Cai, Hongwei Xie

**Affiliations:** Jiangxi Super-Rice Research and Development Center, Jiangxi Provincial Key Laboratory of Rice Germplasm Innovation and Breeding, National Engineering Research Center for Rice, Jiangxi Academy of Agricultural Sciences, Nanchang 330200, China

**Keywords:** *Oryza rufipogon*, *orf182*, wild rice, CMS, structural variation

## Abstract

The widespread adoption of hybrid rice has played a pivotal role in ensuring food security in China. However, the heavy reliance on wild-abortive (WA) cytoplasmic male sterility (CMS) systems raises potential biosafety concerns. In this study, we screened a global collection of wild rice (*Oryza rufipogon*) accessions using *orf182*-specific molecular markers to characterize the geographic distribution patterns of this gene. Mitochondrial sequencing and assembly of 11 representative wild rice species harboring *orf182* revealed 16 novel genes. A total of 469 mitochondrial genes were classified into 23 gene families, with nine families containing single-copy homologous genes, indicating significant gene duplication in mitochondria. We observed a strong positive correlation between mitochondrial genome size and the quantity and size of repetitive sequences. Collinearity analysis revealed extensive mitochondrial variation and large-scale inversions in Guangdong wild rice. Comparative genome analysis uncovered inversions, translocations, and several variations surrounding *orf182*, with a 71 bp repeat sequence mediating the formation of the *orf182*-*nad6* chimeric gene. Gene copy number analysis (GCNV) revealed variable *orf182* gene copy counts (1, 2, and 3) in wild rice species. Additionally, successful transformation of *orf182* from various sources into sterile lines was achieved. These findings provide valuable resources for advancing hybrid rice development in China, thus contributing to enhanced food security.

## 1. Introduction

Mitochondria, often termed the powerhouses of eukaryotic cells, are semi-autonomous organelles believed to have originated from prokaryotic ancestors via endosymbiosis. They harbor their own genetic material, which is indispensable for mitochondrial and cellular functions [1]. The mitochondrial genome (mitochondrial genomes or mtDNA) is typically maternally inherited and follows evolutionary trajectories distinct from those of nuclear genomes. Outbreeding-mediated reshuffling of nuclear and cytoplasmic genomes occurs widely and frequently, potentially reconfiguring the functional and structural interplay between these genetic systems. Rearrangements, mutations, and inheritance patterns in mitochondrial genomes can provoke evolutionary conflicts that significantly influence plant diversification, domestication, and hybridization [2,3]. Nevertheless, the evolutionary mechanisms shaping cytoplasmic genomes—especially among wild relatives of crops—remain inadequately explored.

Structural variation (SV) is considerably more prevalent in mitochondrial genomes than in chloroplast genomes, where SVs are rare and occur at low frequency [4], whereas mitochondrial genomes exhibit extensive SVs driven by frequent repeat-mediated recombination [5]. In contrast, plant mitochondrial genomes undergo extensive recombination mediated by repetitive sequences, leading to pronounced structural diversity [6] and can disrupt gene expression through the reshuffling of regulatory elements or the creation of chimeric open reading frames (ORFs). Many such chimeric ORFs have been associated with cytoplasmic male sterility (CMS), a phenotype characterized by aberrant floral development and abortion of male gametophytes [7,8].

Recombination events are often facilitated by short repeats (SRs; 50–500 bp) and micro-repeats (MRs; <50 bp), which promote asymmetric and largely irreversible recombination, yielding recombinant molecules present at low (sub-stoichiometric) levels [9,10]. These molecules, termed sublimons, can rapidly amplify under certain conditions through a mechanism known as sub-stoichiometric shifting (SSS), thereby altering the mitochondrial genotype (mitotype) and driving mtDNA evolution [11].

Collectively, recombination processes mediated by SRs, MRs, intracellular gene transfer (IGT), and horizontal gene transfer (HGT) generate diverse SVs—including rearrangements, duplications, insertions, and deletions [5,10,12]. Although SVs within gene regions are generally deleterious and selected against, those in intergenic regions may persist, contributing to the high sequence divergence observed in mitochondrial intergenic spacers [13,14,15].

Wild rice (*Oryza rufipogon*), the direct progenitor of cultivated rice (*Oryza sativa*), was domesticated approximately 8000–10,000 years ago. It represents a critical genetic resource for improving stress tolerance, yield, and grain quality in modern rice breeding [16]. While the nuclear genome of *O. rufipogon* has been extensively studied, providing key insights into rice evolution and domestication [17,18], research on its mitochondrial genome remains limited. Early studies using restriction fragment length polymorphism (RFLP) analysis revealed mtDNA variation and distinct mitotypes among wild and cultivated rice populations [19]. Furthermore, several CMS types—including the widely used WA-, BT-, HL-, and D1-CMS systems—have been identified in cultivated rice, many tracing back to *O. rufipogon* or closely related accessions [7,20,21,22]. However, only a few complete mitochondrial genomes of *O. rufipogon* have been reported to date, and a comprehensive comparative analysis is still lacking. As a result, the extent of mitochondrial genomic diversity and its evolutionary relationship with cultivated rice remain poorly understood.

In this study, we employed D1-CMS-type wild rice as a model to investigate cytoplasmic mitochondrial genome evolution. We constructed a pan-mitochondrial genome based on high-quality long-read assemblies from 11 representative accessions of common wild rice. Our comprehensive analyses revealed extensive mitochondrial rearrangements and the presence of chimeric ORFs. Furthermore, we examined the recombination dynamics around the mitochondrial gene *orf182* to elucidate its structural complexity and explore the mechanisms underlying rice mtDNA evolution. Finally, we characterized structural variations across the mitochondrial genome to uncover general architectural features and evolutionary constraints.

## 2. Results

### 2.1. Phylogenetic Tree Constructed by Mitotype-Specific Sequence (MSS)

**Figure 1:**
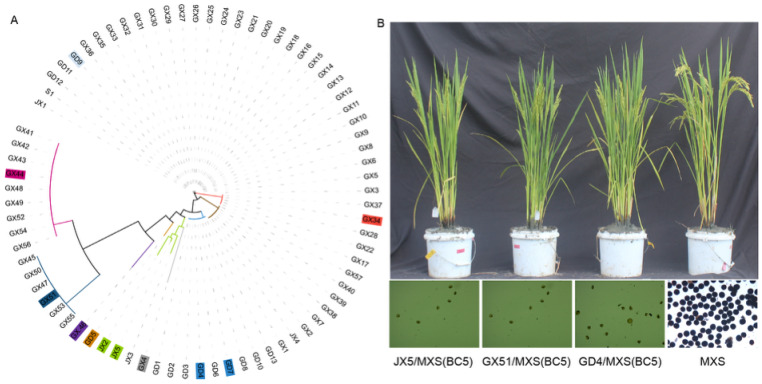
Phylogenetic and habitat-based applications of *Oryza rufipogon* (containing *orf182*). (**A**) Phylogenetic tree constructed by 18 MSSs; the samples in different clades masked by colors were selected for sequencing. (**B**) Three wild rice (*O. rufipogon*) accessions collected from separate provinces in China representing different D1 type variations served as the cytoplasm sources for breeding male-sterile lines. MXS: Mingxiangsi (WA type restorer line).

To further elucidate the evolutionary origin and distribution of this gene, we collected 320 common wild rice accessions from three provinces in China (Guangdong, Jiangxi, and Guangxi), of which 76 (23.75%) carried the D1-type CMS gene orf182 and were retained for subsequent analyses. Phylogenetic reconstruction using 18 highly polymorphic markers [23] resolved the evolutionary relationships within the D1-type wild rice group into distinct clades (Figure 1, Tables S2 and S3). Eleven representative accessions spanning nine phylogenetic clades were selected for mitochondrial genome assembly; comparative analysis of these high-quality assemblies revealed extensive structural variation and recombination events, and highlighted the potential functional role of *orf182* in CMS. Based on three *O. rufipogon* accessions carrying *orf182* from geographically and phylogenetically distinct backgrounds, male sterile lines of Mingxiangsi were successfully obtained after only five backcross generations (Figure 1B), demonstrating that the D1-type CMS system can be efficiently transferred into diverse genetic backgrounds.

### 2.2. General Features of the Oryza rufipogon Mitochondrial Genome

**Figure 2:**
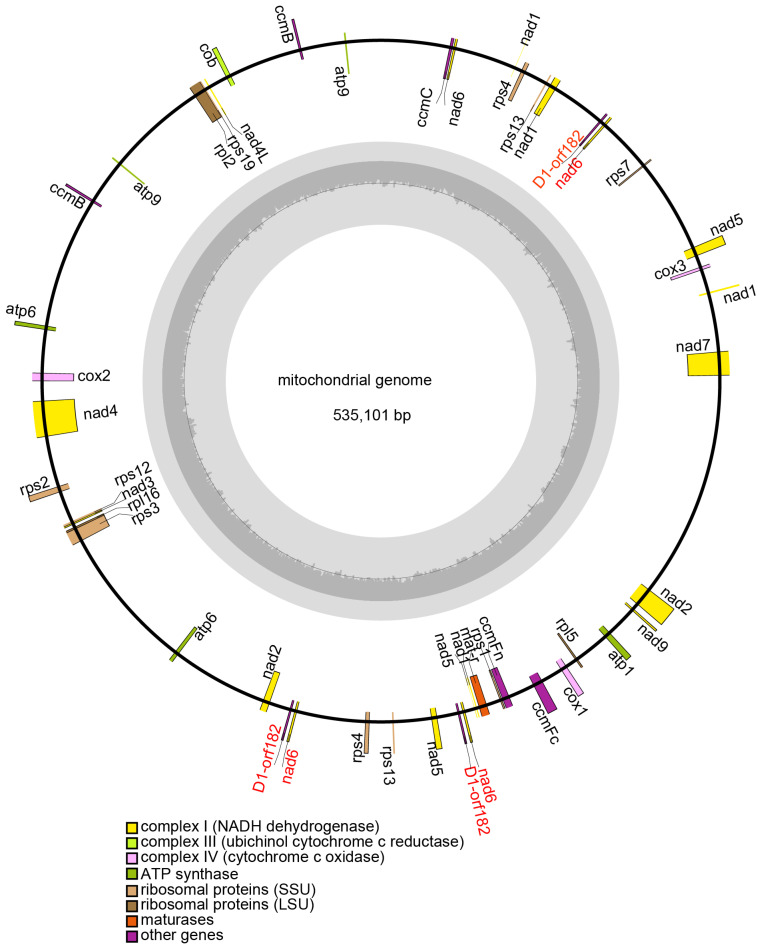
The features of the wild rice JX2 mitochondrial genome. The outward-facing text on the outer circle represents forward-oriented genes, while the inward-facing text denotes reverse-oriented genes. Labels in red indicate the chimeric gene *orf182-nad6*.

A total of 69 Gb of raw Illumina reads (150 bp in length) from 11 samples, along with 506 Mb of Nanopore PromethION data from nine wild rice varieties and 572 Mb of PacBio long reads (average length ~10 kb), were generated for mitochondrial genome assembly (Table S4). The assembled mitochondrial genomes of the 11 wild rice accessions ranged in size from 438,632 bp to 718,357 bp. Functional annotations and physical locations of genes of JX2 are shown in Figure 2, and all 11 wild rice mitochondrial genomes are summarized in Figure S1.

In the JX2 mitochondrial genomes, 77 genes were annotated, including 46 protein-coding genes, 28 tRNAs, and 3 rRNAs. These encode 37 distinct proteins, with *atp4*, *atp6*, *atp8*, *atp9*, *cob-like*, *orf182*, and *orfX* present in two copies each, and *nad6* in three copies. The protein-coding genes were classified into 11 functional categories: ATP synthases (5 genes), cytochrome C biogenesis (4 genes), ubiquinol cytochrome c reductase (1 gene), cytochrome c oxidase (3 genes), maturases (1 gene), NADH dehydrogenase (9 genes), cytoplasmic male sterility-related (1 gene), open reading frames (1 gene), large ribosomal subunit proteins (3 genes), and small ribosomal subunit proteins (8 genes).

On average, seven chimeric ORFs derived from fragments of known and conserved protein-coding genes (excluding tRNAs and rRNAs) were identified in each mitochondrial genome. Notably, *nad6* (from the *nad* family) and *rpl2* and *rpl16* (from the *rpl* family) frequently participated in the formation of prominent chimeric ORFs across all wild rice accessions examined (Table S5).

To assess structural variation (SV) and its functional implications, we performed gene copy number analysis. A core set of 37 protein-coding genes was conserved across all 11 mitochondrial genomes. Among these, *nad6* exhibited the highest copy number. The accession GX34 showed the most extensive gene loss, affecting *atp4*, *atp8*, *rps7*, *cox3*, *nad5*, *nad7*, *rps13*, *ccmFc*, *rpl5*, and *cox1*. Additionally, genes such as *cob-like* and *orfX* were lost in two other accessions. Gene duplication was widespread: *Cox1* was duplicated in 8 out of the 10 accessions in which it was present, and *nad6* duplication occurred in all 11 accessions (Figure S2).

### 2.3. Repeat Sequence Variation and Correlations Between Genome Size and Repeat Sequences

**Figure 3:**
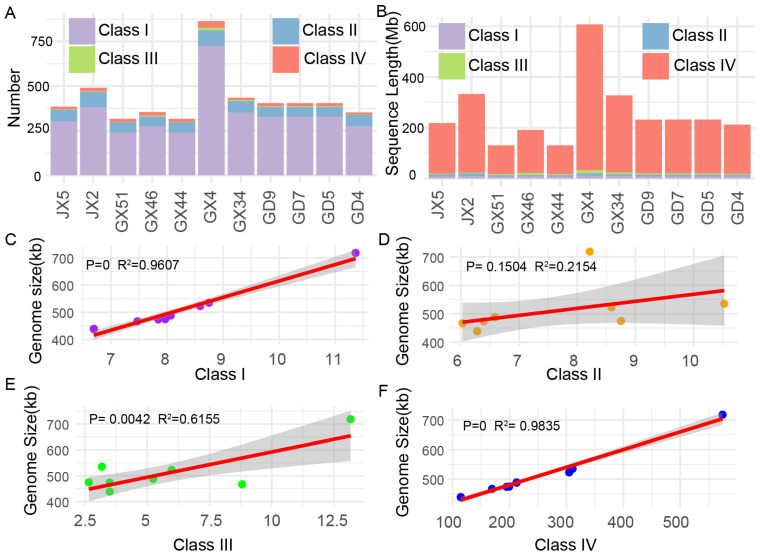
Repeat content in D1-type wild rice mitochondrial genome. (**A**,**B**) Total number and length of different repeat types in 11 wild rice accessions mitochondrial genomes. Class I: <100 bp, class II: (100 bp, 500 bp), class III: (500 bp, 2000 bp), class IV: >2000 bp. (**C**–**F**) The correlation between genome size and different repeat type number (**left**), total repeat length. Each point represent a sample. R indicates the correlation coefficient, and the *p*-value was determined by a two-tailed Student’s *t*-test.

Mitochondrial genome repeats were classified into class I (repeats ≤ 100 bp), class II (100–500 bp), class III (500–2000 bp), and IV (>2000 bp) based on repeat length. The number of class I repeat sequences ranged from 239 (GX44 and GX51) to 724 (GX4), accounting for 1.53–1.68% of mitochondrial genome size (Figure 3A; Table S6). Among the samples, the number of class I repeats in the GX4 samples was significantly higher than samples from wild rice in the same province and other wild rice, while the number of repeats longer than 100 bp was not significantly increased (*t*-test, *p*-value = 0.11) (Table S6). Meanwhile the total repeat number (864) of GX4 was higher than that of the other samples (total repeat number: 316–490) (Figure 3), and the total repeat length showed a similar pattern, while the mitochondrial size of GX4 was greater than the others contributed by the class IV type repeat. These were followed by repeats in class II, which accounted for approximately 15% of the total repeats, with 56–90 in number. There were the most least repeat lengths located in class III and only accounted for 0.92%. In contrast, a total of 12–38 repeats in class IV were identified, with lengths ranging from 114.38 to 574.38 kb, contributing 26.04–79.96% of the mitochondrial genome size. Despite the relatively low number of repeats in class IV, they were the major contributors to the total mitochondrial genome size (Figure 3B).

For all of the 11 wild rice samples, mitochondrial genome size showed significantly high correlations (*p*-value < 0.001) with the total repeat number and length (Figure S3 and Figure 3C,D). All counts of the four repeat categories showed significant correlation with variations in genome size (R2 adj ranged from 0.63 to 0.99, *p*-value < 0.003) (Figure S3), while the class II repeat size showed low correlations with the mitochondrial genome size (Figure 3D). In addition, several mitochondrial genomes may have an overrepresentation of repeat content (Table S6). For example, GX46 (total repeat length: 466 kb) had a similar genome size to GX51 and GX44 (438 kb), but the total repeat length was about 1.46-fold of GX51 (131 kb).

### 2.4. Identified the Structural Variation in D1-Wild Rice Mitochondrial Genomes

**Figure 4:**
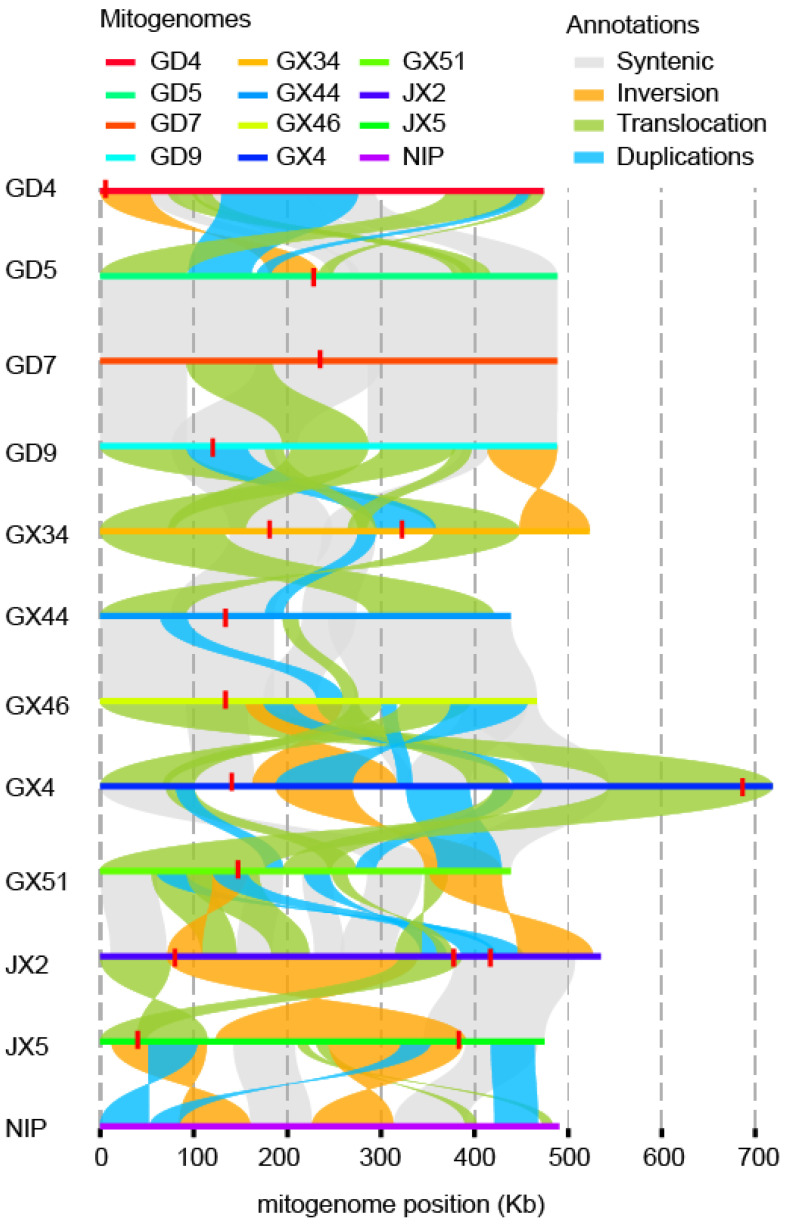
Structural variation between D1-type wild rice and Nipponbare. The red vertical tags on the mitochondrial genome represent *orf182*. Gray lines between two genomes indicate collinearity, while yellow denotes inversions, green represents translocations, and blue corresponds to duplications.

Previous studies have indicated that repeat sequences can mediate rearrangements of varying extent in the mitochondrial genome. By aligning the 11 assembled mitochondrial genomes to the Nipponbare reference genome, we identified syntenic regions accounting for 12% to 30% of each genome (Table S7). Structural variations across assemblies included one to three inversions, two to six translocations, 27 to 68 insertions, and 32 to 61 deletions (Figure 4 and Figure S4). Wild rice accessions with D1-CMS exhibited the highest abundance of SVs (Figure S5). Notably, although GX34 ranked fourth lowest in terms of SV quantity, it displayed the greatest total SV length. This apparent discrepancy is largely attributable to the absence of large-scale translocations in GX34, resulting in a notably low minimal SV length.

Mitochondrial genomes of wild rice from Guangdong Province appeared relatively conserved, a pattern that may reflect either geographic sampling concentration or limited underlying genetic differentiation. In contrast, the two Jiangxi accessions exhibited markedly distinct profiles in both the number and size of SVs (Figure S5).

Note that 15 out of the 17 LCBs are displayed as colored blocks labeled A–N, while the remaining two LCBs are represented solely by syntenic lines due to their highly rearranged or fragmented nature across genomes (Figure S6). Three major rearrangement events were detected in Jiangxi Dongxiang wild rice, whereas only five LCBs were identified in GX4 and GX34. GX34 exhibited extensive gene loss and fewer large-scale structural rearrangements relative to other accessions, indicating a relatively conserved genomic architecture.

### 2.5. Pan-Mitochondrial Genome of 11 Selected D1 CMS Wild Rice and Nipponbare

To further investigate mitochondrial genomic diversity across accessions of different geographical origins, we selected 11 D1-CMS wild rice accessions representing phylogenetically distinct groups, together with the previously published Nipponbare mitochondrial genome, for pan-genome construction and comparative analysis.

**Figure 5:**
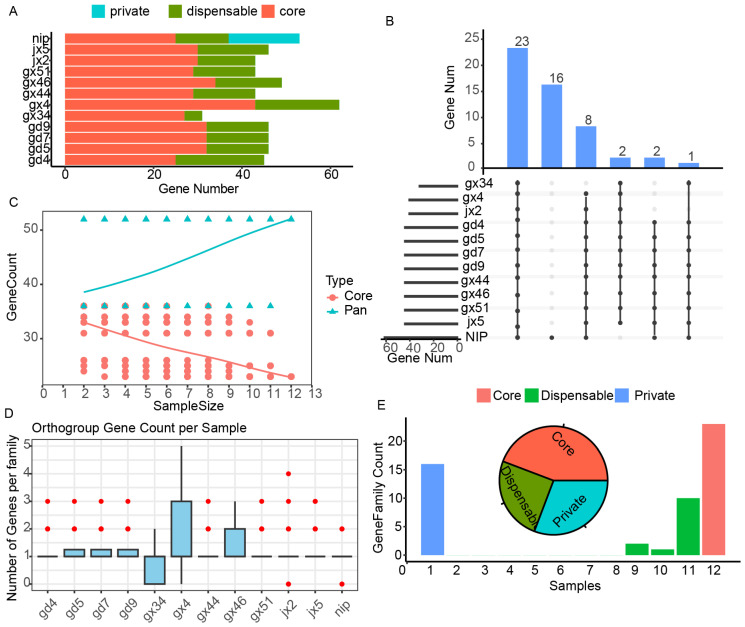
Pan-mitochondrial genome of the 11 D1-type wild rice and Nipponbare. (**A**) Distribution of annotated protein-coding genes across individual accessions, showing the composition of core (red), dispensable (green), and private (blue) gene sets in each accession. X-axis means the gene number, y-axis indicates the wild rice samples. (**B**) UpSet plot showing the intersection and distribution of gene families across accessions. Vertical bars represent the number of gene families shared among specific combinations of accessions, while the connected dots below indicate the corresponding intersection sets. Horizontal bars (**left**) denote the total number of genes in each accession. (**C**) Pan-genome and core-genome size dynamics with increasing sample size. The pan-genome (teal) shows a continuous increase as additional genomes are incorporated, whereas the core genome (red) gradually decreases, indicating progressive reduction in shared genes across all accessions. (**D**) The orthogroup gene count per sample. (**E**) The number and proportion of core, dispensable, and private gene families in the pan mitochondrial genome.

The gene accumulation curve demonstrated a continuously open pan-genome (Figure 5C), indicating that additional mitochondrial genes are likely to be discovered as more rice genomes are sequenced. The upset plot further resolved the intersecting gene sharing patterns across accessions, revealing that 23 genes were shared among all 12 genomes, 16 were shared among a subset of accessions, and 8 among fewer genomes, with progressively fewer genes present in smaller subsets down to singletons (Figure 5B). Notably, private genes, those present in only a single accession, were exclusively detected in the Nipponbare reference genome, with no private genes identified among the 11 D1-CMS wild rice accessions, highlighting substantial divergence in mitochondrial gene content between the cultivated reference and the wild rice lineages examined here (Figure 5A,B).

Geographical patterns in pan-genome composition were evident across accessions. Mitochondrial genomes from the Guangdong accessions were generally the most conserved, however, GD4 was an exception, exhibiting a reduced core gene complement and a higher proportion of dispensable genes. Accessions from Guangxi displayed greater overall variability, although GX34 stood out for its relatively lower dispensable gene content, suggesting a more stable evolutionary trajectory and possible adaptation to a narrower ecological niche. Among the Jiangxi accessions, JX5 harbored more variable genes than JX2, indicating divergent evolutionary histories within the same province (Figure 5A).

At the level of gene family composition, core families accounted for the largest share (44.23%), followed by private (30.77%) and dispensable (25.00%) families. In terms of absolute gene copy number, however, core genes dominated with 66.55% of all copies, while private genes constituted only 2.89%, reflecting the high conservation of shared mitochondrial functions across these genomes (Figure 5E). Analysis of orthogroup gene copy number per sample revealed that most gene families were present as single copies across accessions, consistent with the low redundancy typical of plant mitochondrial genomes. GX4 and GX46 showed slightly elevated copy numbers in certain orthogroups, suggesting local gene duplication or structural expansion events in these lineages (Figure 5D).

## 3. Discussion

In this study, we identified the chimeric mitochondrial gene *orf182* as a unique genetic element present exclusively in a subset of Chinese wild rice accessions. This finding offers significant insights into the evolutionary dynamics of plant mitochondrial genomes, the origin of cytoplasmic male sterility (CMS)-associated genes, and the potential utility of *orf182* in hybrid rice breeding. The restricted distribution of *orf182* suggests that it likely originated from a localized mitochondrial recombination event. This observation supports the hypothesis that CMS-related genes often emerge independently in different populations through rapid and extensive mitochondrial restructuring. Similar to *orf79* and *orfH79*, *orf182* exemplifies the high structural plasticity of plant mitochondrial genomes, wherein recurrent gene fusion events give rise to chimeric open reading frames. The occurrence of *orf182* solely in Chinese wild rice further indicates that CMS genes are not universally conserved but rather represent region-specific evolutionary innovations. Its discovery highlights the distinctive genetic value of Chinese wild rice germplasm within the global rice gene pool. As a naturally occurring CMS-inducing gene, *orf182* provides direct evidence of functional diversity preserved in wild populations. Among the collected Chinese *Oryza rufipogon* accessions, 23.75% were found to carry *orf182*. Based on these findings, we propose that the mitochondrial genome harboring *orf182* has not evolved through conservative mechanisms but rather via dynamic structural rearrangements.

### 3.1. Mitochondrial Genome Structural Plasticity and Chimeric Gene Formation

**Figure 6:**
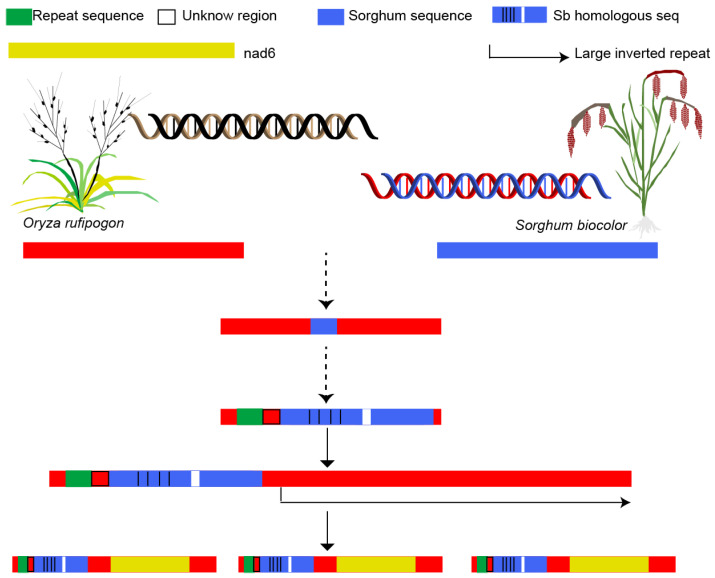
The evolutionary trajectory of *orf182* in rice. Green indicates 71 bp repeat sequence shared by mitochondrial and nuclear genome. Empty rectangular area means unknown region, blue rectangle shows the sequence derived from the *Sorghum biocolor* mitochondrial genome. The dashed line indicates an unknown mechanism. Blue rectangle with black vertical line (SNP) and white region (InDel) denotes sequence variation compared to the *Sorghum biocolor* sequence, yellow rectangle stands for the *nad6* gene, and the hairpin arrow indicates a large inverted repeat region.

Our results demonstrate that large-scale structural variations (LSVs) are prevalent in the mitochondrial genomes of wild rice, including among accessions from the same geographic region. Accessions such as GD4 and GX4 exhibit pronounced structural divergence compared to others. Notably, the physical scale of SVs did not correlate with their quantity; for instance, GX34 displayed the largest SV size but ranked among the lowest in terms of SV number. Substantial variation in mitochondrial genome size was observed across the wild rice panel (Figure 3). The assembly and annotation of 11 mitochondrial genomes provide new insights into this variability. None of the wild rice accessions shared a common presence–absence variation (PAV) region associated with *orf182*, indicating that this gene did not originate from a recent insertion event. Instead, analysis of repetitive sequences revealed that some *orf182* chimeric genes likely arose from duplication events, while others were generated through recombination mediated by a 71-bp repetitive element, which appears to have undergone multiple recombination cycles (Figure 6).

### 3.2. CMS Gene Diversity and Implications for Domestication and Hybrid Breeding

Dynamic structural changes, including gene duplications, have facilitated the emergence of new gene copies in the mitochondrial genome. Nucleotide alignment of *orf182* sequences identified several SNP sites, which were categorized into three haplotypes (Figure S7). All detected SNPs were synonymous, suggesting neutral evolution at the sequence level. CMS genes such as *orf182* may have historically functioned as genetic barriers that contributed to reproductive isolation, thereby influencing domestication pathways and exhibiting geographic specificity. The D1-CMS system, characterized by *orf182*, represents a promising resource for the development of novel hybrid rice breeding systems.

## 4. Material and Methods

### 4.1. Plant Materials

*Oryza rufipogon* collected from three provinces, Guangdong (GD), Guangxi (GX), and Jiangxi (JX), was used in the present research. The Guangdong common wild rice materials were generously contributed and identified by Professor Yonggen Lu (South China Agricultural University); Guangxi wild rice was obtained from researcher Yuntao Liang (Guangxi Academy of Agricultural Sciences); while Jiangxi wild rice was kindly provided by the Dongxiang Wild Rice Resource Nursery and identified by Hongwei Xie. *Oryza spontanea* (N104625) was provided by the International Rice Research Institute, Philippines. The voucher specimens (refer to Table S1) are stored in the herbarium of Jiangxi Academy of Agricultural Sciences, and relevant data are stored in the Germplasm Resource Database of the Jiangxi Academy of Agricultural Sciences (currently an internal website, and are available from the corresponding author upon reasonable request). All the wild rice and *O. spontanea* were grown in the Wild Rice Germplasm Resource Nursery of the Jiangxi Academy of Agricultural Sciences (28°56′20″ N, 115°39′59″ E).

### 4.2. Construction of Maximum Likelihood Tree Based on the Mitotype-Specific Sequences (MSSs)

To select the representative D1-CMS relatives among the 76 common wild rice collected worldwide, the previously developed 20 pairs of evenly distributed D1-CMS mitochondrial specific sequences were used (Table S2). The 0/1 matrix was generated by the MTTs gel results (Table S3). The phylogenetic tree was constructed using the maximum likelihood method in IQ-TREE software v1.6.12 [24], and the resulting maximum likelihood tree was visualized using ITOL online tools (iTOL: Interactive Tree Of Life (https://itol.embl.de/), 18 July 2025) [25].

### 4.3. Samples, DNA Extraction and Mitochondrial Genome Sequencing

A total of 11 representative wild rice accessions were selected based on the phylogenetic tree constructed using MSSs in this study (Table 1). Fresh leaves were collected from plants grown under field conditions at the Jiangxi Academy of Agricultural Sciences, Nanchang, China. High-molecular-weight (HMW) genomic DNA was extracted from 1-month-old leaves following the standard cetyltrimethylammonium bromide (CTAB) method, with DNA quality and quantity assessed by agarose gel electrophoresis and a NanoDrop spectrophotometer prior to library preparation.

Mitochondrial genomes of all 11 wild rice accessions were sequenced using three complementary platforms to enable hybrid assembly. For short-read sequencing, paired-end libraries with an insert size of ~300 bp were constructed following Illumina’s standard genomic DNA library preparation protocol. Libraries were sequenced on the Illumina NovaSeq 6000 platform (2 × 150 bp) at Shanghai BIOZERON Co., Ltd. (Shanghai, China). For long-read sequencing, HMW DNA was used to prepare SMRTbell libraries following the PacBio standard protocol (Pacific Biosciences, Menlo Park, CA, USA) and sequenced on the PacBio Sequel II system to generate CCS (HiFi) reads with a mean accuracy > 99%. Additionally, Oxford Nanopore libraries were prepared using the Ligation Sequencing Kit (SQK-LSK110; Oxford Nanopore Technologies, Oxford, UK) and sequenced on a GridION device to produce ultra-long reads for gap resolution and repeat region spanning. Raw reads from all three platforms were subjected to quality control prior to genome assembly.

### 4.4. Mitochondrial Genome Assembly and Annotation

Raw sequencing reads were trimmed using Trimmomatic (version 0.35) to remove low-quality bases (quality score < 20) and adapter sequences, retaining reads with a minimum length of 50 bp. High-quality reads were assembled de novo using two complementary assemblers—SOAPdenovo2 v2.04 and Celera Assembler v8.0—with k-mer sizes optimized for each dataset. Scaffolds were constructed using SSPACE (v3.0; default parameters), and remaining gaps within scaffolds were filled using GapCloser v1.12 with default parameters [26]. Redundant or duplicated sequences were identified by self-alignment (BLASTn, identity ≥ 95%, coverage ≥ 90%) and removed to produce the final, non-redundant mitochondrial genome assembly for each accession.

Genome annotation was performed by integrating de novo prediction with homology-based approaches. For homology-based annotation, protein sequences from the *Oryza sativa* ssp. japonica cv. Nipponbare reference mitochondrial genome (GenBank: BA000029.3) were aligned to each assembled genome using BLAST v2.14 (E-value ≤ 1 × 10^−5^), followed by precise spliced alignments using GeneWise v2.4.1 to delineate exon–intron boundaries. Alignments with identity < 70% or coverage < 50% were excluded. De novo gene prediction was conducted using AUGUSTUS (http://bioinf.uni-greifswald.de/augustus/, 18 July 2025) with parameters trained for plant mitochondrial genomes. Transfer RNA genes were identified using tRNAscan-SE v2.0 (default parameters, eukaryote model). All gene models were integrated and weighted using EVidenceModeler v1.1.1 [27], with priority given to homology-based evidence. The resulting GenBank-format annotation files were manually reviewed and curated. Circular mitochondrial genome maps were drawn using OGDRAW (https://chlorobox.mpimp-golm.mpg.de/OGDraw.html, 19 December 2025).

### 4.5. Analysis of Repeated Sequences

Interspersed and large-fragment intragenomic repeats were identified using BLASTn with an E-value threshold of 1 × 10^−5^, a minimum identity of 90%, and a minimum alignment length of 100 bp; self-hits and overlapping alignments were excluded. Simple sequence repeats (SSRs/microsatellites) were detected using MISA (MIcroSAtellite identification tool) with the following minimum repeat unit thresholds: mononucleotide (≥8 repeats), dinucleotide (≥4 repeats), trinucleotide (≥4 repeats), tetranucleotide (≥3 repeats), pentanucleotide (≥3 repeats), and hexanucleotide (≥3 repeats). Tandem repeats with period size ≤ 500 bp were detected using Tandem Repeats Finder v4.10 [28], retaining only those with more than 7 repeat units. Direct and inverted repeats were identified using REPuter (https://bibiserv.cebitec.uni-bielefeld.de/reputer/, 3 November 2025) with a minimum repeat size of 20 bp, a Hamming distance ≤ 3, and an E-value threshold of 1 × 10^−5^ to control for false positives.

### 4.6. Collinear Analysis and Comparative Analysis Among the D1-CMS Wild Rice

The *O. sativa* ssp. *japonica* cv. Nipponbare mitochondrial genome characterized by Notsu et al. [29] (GenBank: BA000029.3) was used as the reference for all comparative analyses. Pairwise collinearity between each wild rice mitochondrial genome and the Nipponbare reference was assessed using MUMmer v3.23 [30]. Syntenic blocks were identified using the nucmer module under many-to-many alignment mode with a minimum identity threshold of 95% and a minimum cluster length of 500 bp; matches shorter than 100 bp were discarded using the delta-filter utility. Collinearity maps were visualized using Plotsr as implemented within the SYRI framework [31].

Genome-level attributes, including assembly size, GC content, nucleotide base composition, start and stop codon usage, total gene counts, and intron numbers, were compiled across all 11 wild rice mitochondrial genomes and compared to the Nipponbare reference to characterize inter-accession variation. The copy number of each protein-coding gene within individual mitochondrial genomes was determined by BLASTn-based self-comparison (identity ≥ 95%, coverage ≥ 90%).

The genome sizes, GC contents, base compositions, start and stop codons, gene counts, and intron numbers of 11 wild rice mitochondrial genomes were compared to assess their variations and similarities. The copy number of protein coding genes among the mitochondrial genome were identified.

### 4.7. Rearrangement Event Identification in D1-Type Wild Rice Mitochondrial Genomes

To systematically identify structural rearrangements among the wild rice mitochondrial genomes, whole-genome multiple alignments were performed using Mauve v2.4.0 [32], which detects locally collinear blocks (LCBs) and rearrangement events including inversions and translocations. Mitochondrial genome assemblies from all 11 wild rice accessions, representing distinct geographic regions across China, were aligned using default progressive Mauve parameters. LCBs shared across genomes were identified from the alignment output, and regions exhibiting altered block order or orientation were flagged as candidate rearrangement sites.

Rearrangement events were classified as follows: inversions were defined as LCBs displaying reversed orientation relative to the reference, while translocations were inferred when LCBs occupied non-syntenic positions relative to their expected genomic coordinates. To minimize false positives arising from small repetitive regions, only LCBs exceeding 1 kb in length were retained for downstream analysis. Candidate rearrangement regions were cross-validated against repeat sequence annotations (Section 4.5), given that repeat-mediated recombination is a principal driver of plant mitochondrial genome structural variation. The spatial overlap between rearrangement breakpoints and annotated repeat elements was assessed to infer potential mechanistic drivers of structural diversification.

### 4.8. Construction of Mitochondrial Genome Variant Maps

Single nucleotide polymorphism (SNP) and insertion/deletion (INDEL) calling across all 11 D1-type wild rice accessions was performed using the Nipponbare mitochondrial genome (GenBank: BA000029.3) as the reference. Raw reads were quality-trimmed using Trimmomatic v0.39 [33] with the following parameters: ILLUMINACLIP (adapter removal, 2:30:10), LEADING:3, TRAILING:3, SLIDINGWINDOW:4:20, and MINLEN:50. Clean reads were mapped to the reference using BWA-MEM v0.7.17 [34] with default parameters. SAM files were converted to sorted BAM format using SAMtools v1.12 [35], and PCR duplicates were marked and removed using Picard v2.25.0 MarkDuplicates (https://broadinstitute.github.io/picard/, 3 November 2025). Variant calling was performed using GATK v4.1 HaplotypeCaller in GVCF mode [36], followed by joint genotyping with GenotypeGVCFs. Variant filtration was applied using GATK (version 4.1) VariantFiltration with the following hard-filter thresholds for SNPs: QD < 2.0, FS > 60.0, MQ < 40.0, MQRankSum < −12.5, and ReadPosRankSum < −8.0; for INDELs: QD < 2.0 and FS > 200.0. Final variant sets were filtered to retain only sites with a minor allele frequency (MAF) > 0.05 and a missing data rate < 10% across accessions.

### 4.9. Analyses of the Protein-Coding-Gene Based Pan-Mitochondrial Genome

To construct a pan-mitochondrial genome based on protein-coding gene content, the predicted peptide sequences of all protein-coding genes from all 12 mitochondrial genomes (11 wild rice accessions plus the Nipponbare reference) were compared in an all-versus-all manner using BLASTp (E-value ≤ 1 × 10^−10^, identity ≥ 30%, coverage ≥ 50%). Orthologous gene clusters were identified from the BLAST results using OrthoFinder v2.5 [37] with default parameters (inflation value = 1.5). Gene clusters were then classified into three pan-genome categories: (i) the core genome, comprising gene families present in all 12 mitochondrial genomes; (ii) the dispensable genome, comprising gene families present in 2 to 11 mitochondrial genomes; and (iii) the private (accession-specific) genome, comprising gene families found in only a single mitochondrial genome. The size and composition of each pan-genome compartment were summarized and compared across accessions.

## Figures and Tables

**Table 1:** The representative 11 wild rice types selected for mitochondrial genome assembly.

Series Number	Accession Number	Species	Origin (Province)	Voucher Specimens
GX4	802	*O. rufipogon*	China (Guangxi)	JXSNKY-GX-0004
GX34	N567	*O. rufipogon*	China (Guangxi)	JXSNKY-GX-0034
GX44	K40	*O. rufipogon*	China (Guangxi)	JXSNKY-GX-0044
GX46	K70	*O. rufipogon*	China (Guangxi)	JXSNKY-GX-0046
GX51	K130	*O. rufipogon*	China (Guangxi)	JXSNKY-GX-0051
GD4	GW8	*O. rufipogon*	China (Guangdong)	JXSNKY-GD-004
GD5	GW11	*O. rufipogon*	China (Guangdong)	JXSNKY-GD-005
GD7	GW14	*O. rufipogon*	China (Guangdong)	JXSNKY-GD-007
GD9	GW22	*O. rufipogon*	China (Guangdong)	JXSNKY-GD-009
JX2	DX50	*O. rufipogon*	China (jiangxi)	JXSNKY-JX-002
JX5	DX219	*O. rufipogon*	China (jiangxi)	JXSNKY-JX-005

## Data Availability

The mitochondrial genome sequences of the long reads and short reads supporting this study have been submitted to the National Center for Biotechnology Information (NCBI) with BioProject ID: PRJNA1338361 and PRJNA1338456. The materials that support the findings in this study are available from the corresponding author, Hongwei Xie, upon reasonable request.

## Supplementary Materials

The following supporting information can be downloaded at: https://www.mdpi.com/article/10.3390/plants15071111/s1.
