# Supplementary figures and images for: Comprehensive Analysis of Wild Rice Mitochondrial Genomes Reveals Structural Variation, Repeat Dynamics, and the Evolution of orf182
Source: Plants (Basel). 2026 Apr 3;15(7):1111. doi: 10.3390/plants15071111 (PMC13074710; doi:10.3390/plants15071111)

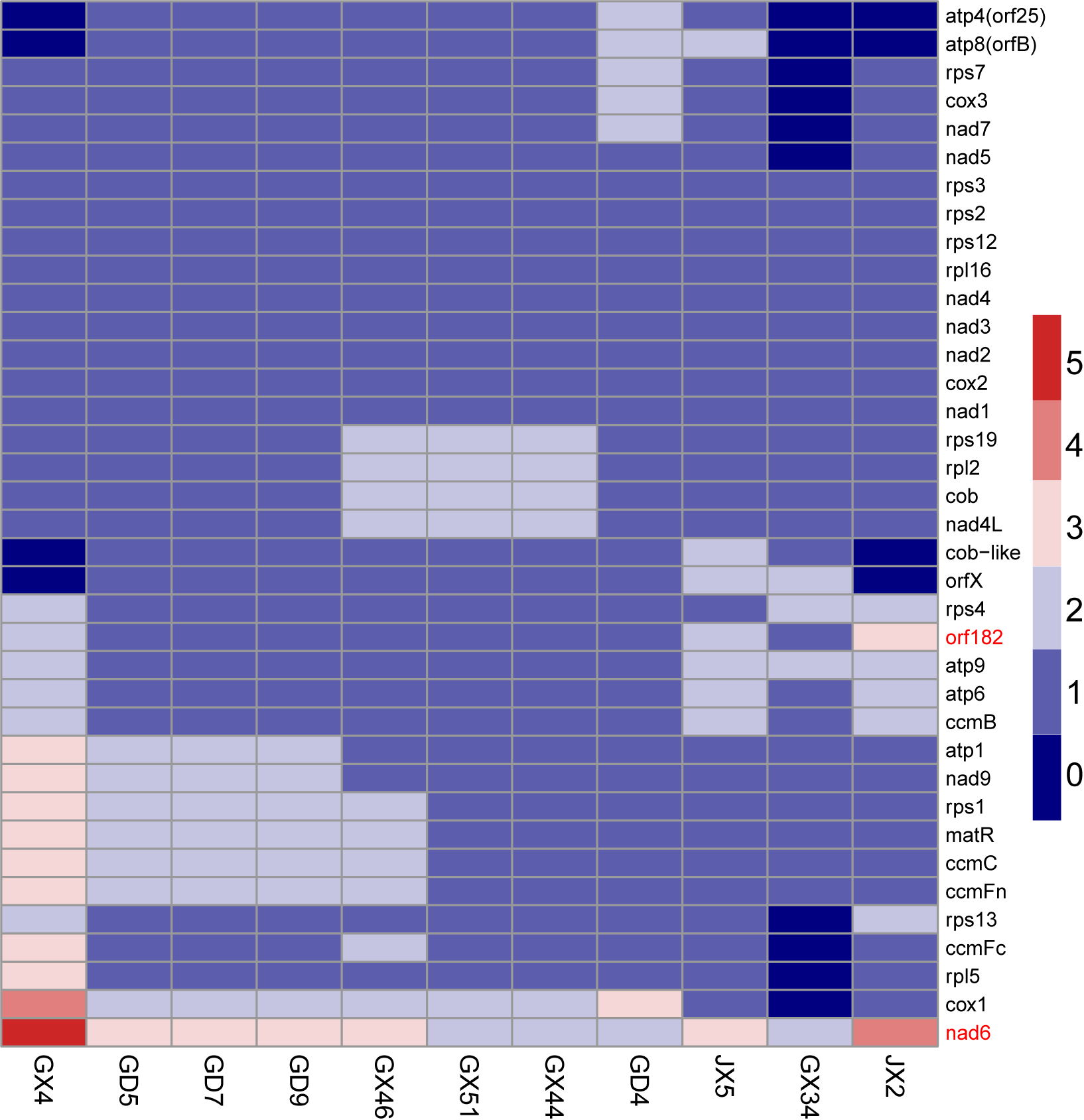

Supplement: Supplementary file 1 [file plants-15-01111-s001.zip › fig S2 gene repeat.tif]

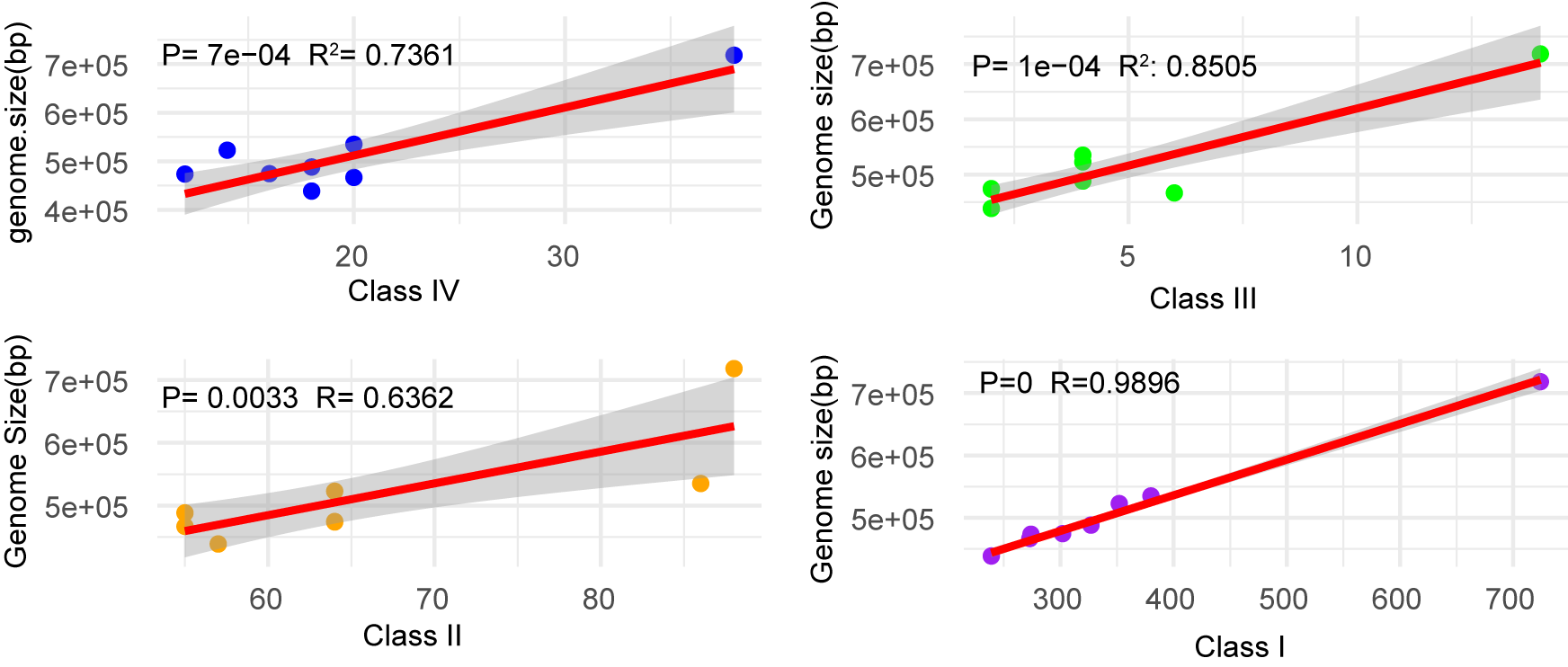

Supplement: Supplementary file 1 [file plants-15-01111-s001.zip › Fig S3 Repeat_count.tif]

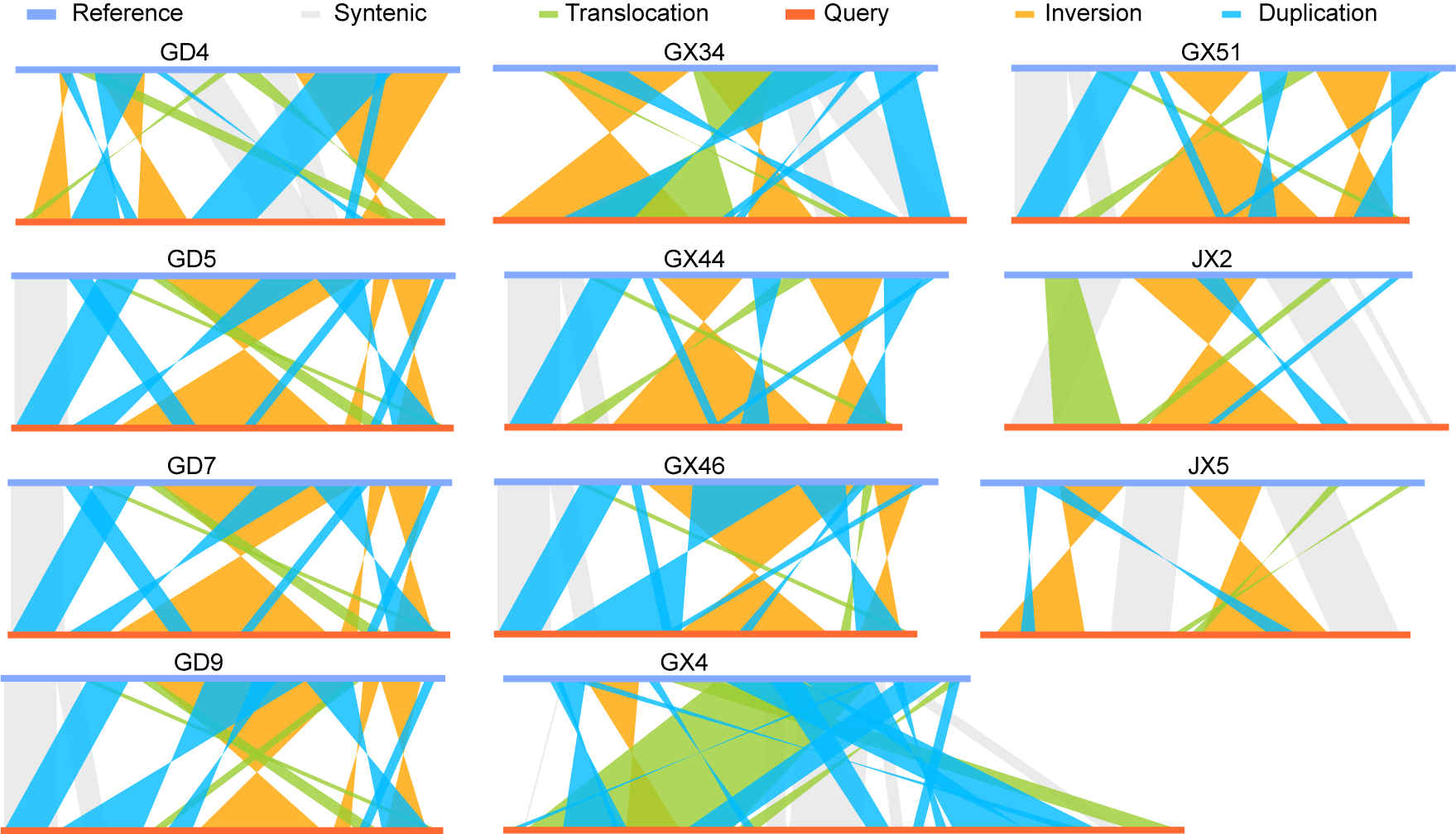

Supplement: Supplementary file 1 [file plants-15-01111-s001.zip › fig S4 syri summary.tif]

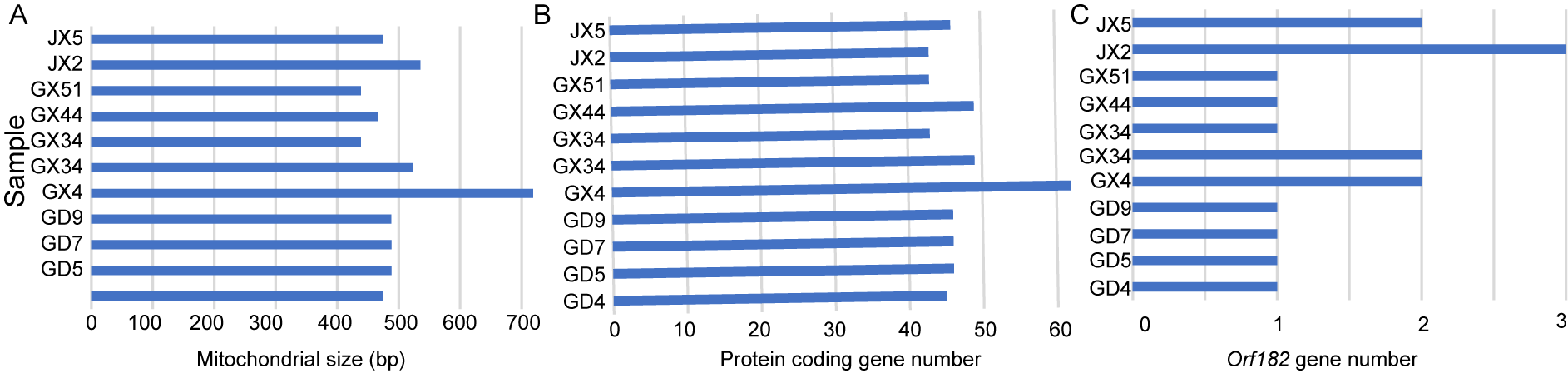

Supplement: Supplementary file 1 [file plants-15-01111-s001.zip › figure S1.tif]

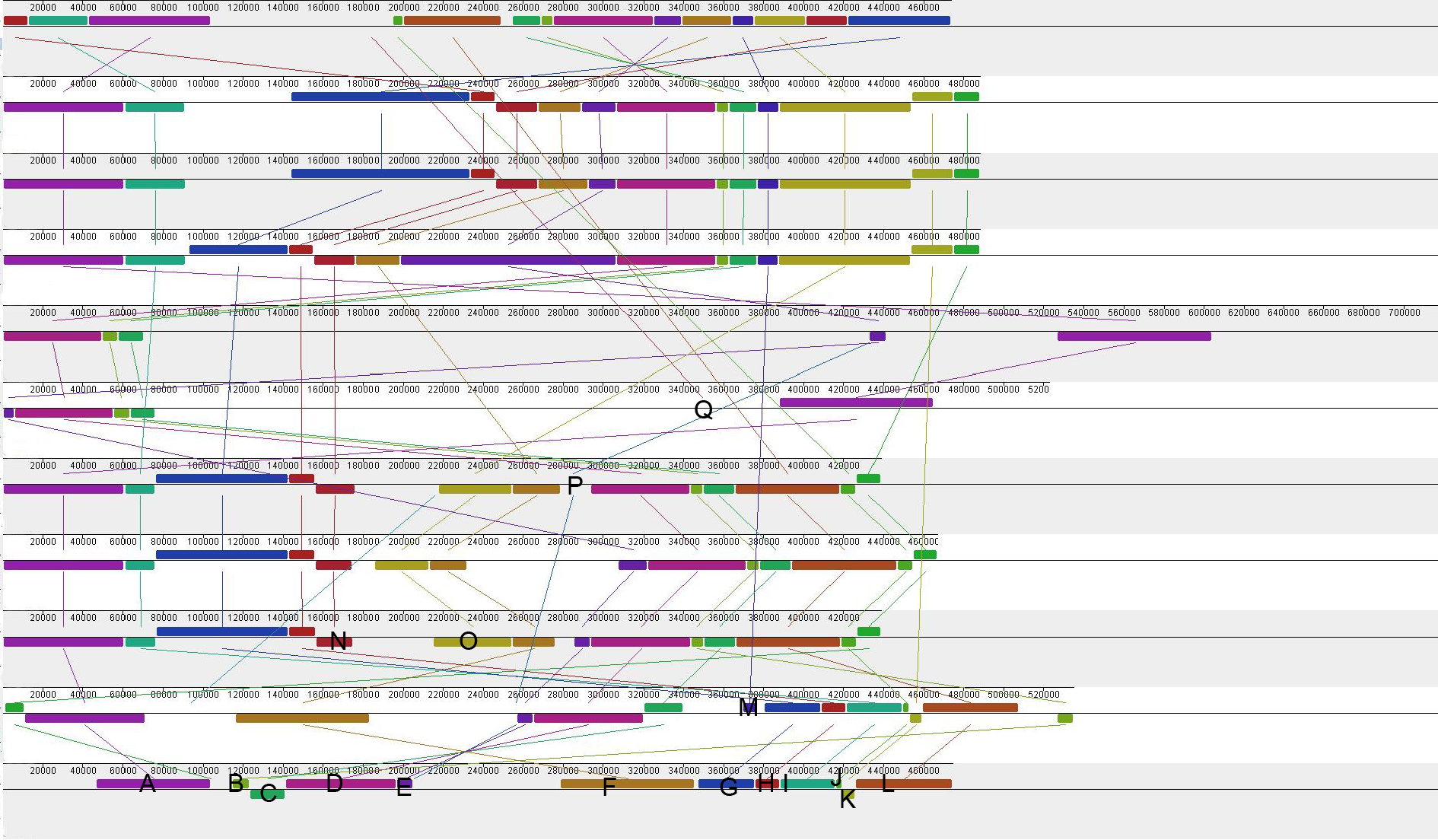

Supplement: Supplementary file 1 [file plants-15-01111-s001.zip › figure S5.tif]

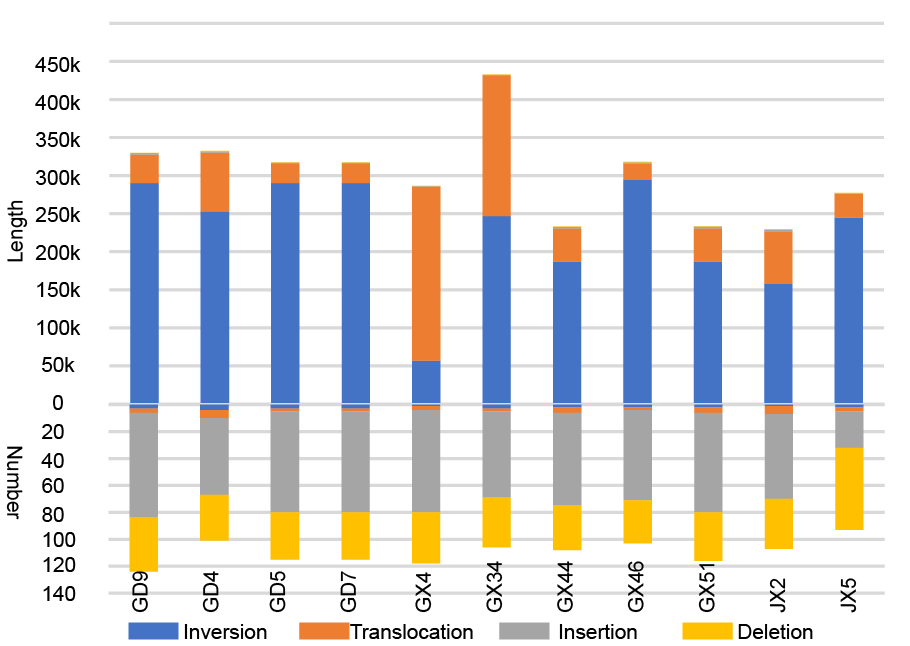

Supplement: Supplementary file 1 [file plants-15-01111-s001.zip › Figure S6.tif]

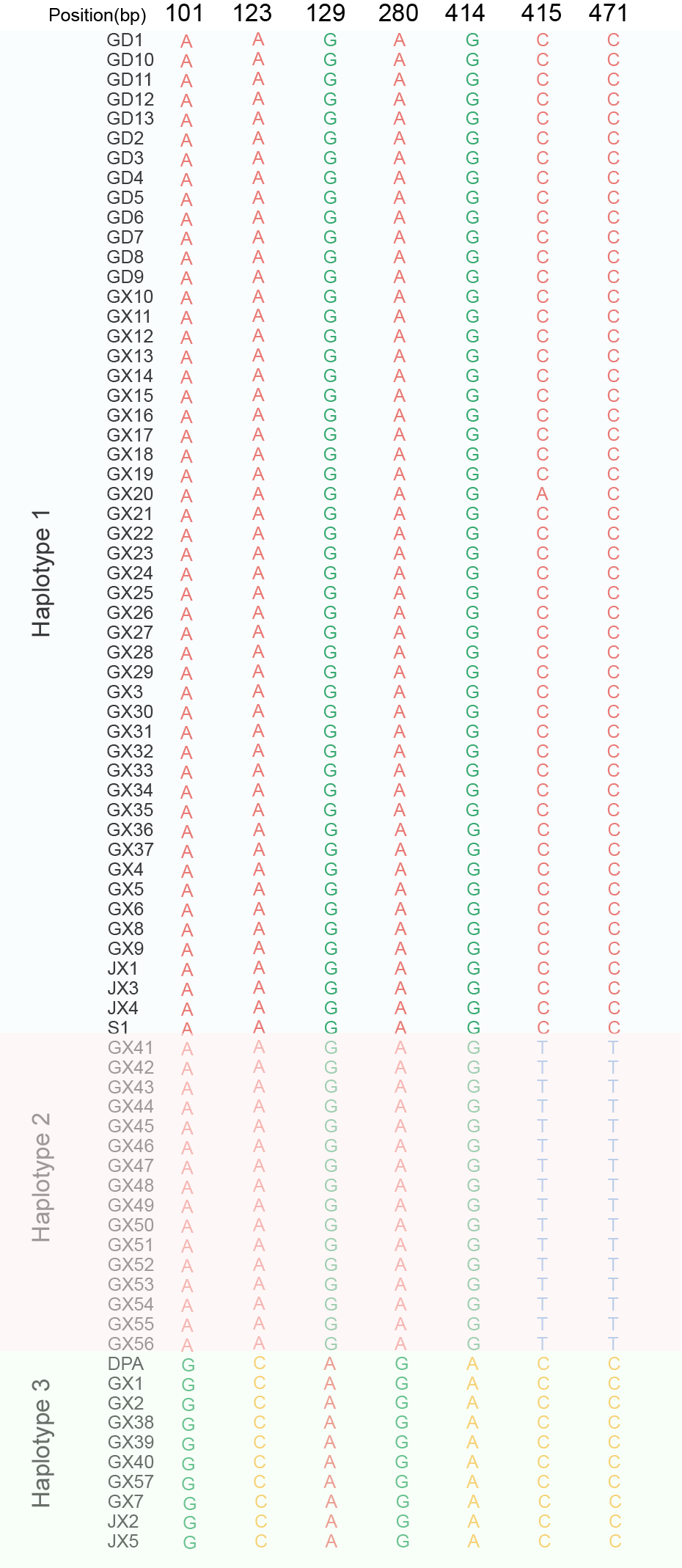

Supplement: Supplementary file 1 [file plants-15-01111-s001.zip › Figure S7.tif]
